# The role of multilateral organizations and governments in advancing social innovation in health care delivery

**DOI:** 10.1186/s40249-019-0592-y

**Published:** 2019-09-13

**Authors:** Beatrice Halpaap, Rosanna W. Peeling, François Bonnici

**Affiliations:** 1 UNICEF/UNDP/World Bank/WHO Special Programme for Research and Training in Tropical Diseases (TDR), World Health Organization, Geneva, Switzerland; 2 London School of Hygiene and Tropical Medicine, London, UK; 3 Bertha Centre for Social Innovation & Entrepreneurship, University of Cape Town, Cape Town, South Africa

**Keywords:** Social innovation, Health care delivery, Community engagement, Multidisciplinary research, Multilateral organizations, Government engagement

## Abstract

**Background:**

Despite great medical advances and scientific progress over the past century, one billion people globally still lack access to basic health care services. In the context of the 2030 Agenda for Sustainable Development social innovation models aim to provide effective solutions that bridge the health care delivery gap, address equity and create social value. This commentary highlights the roles of multilateral organizations and governments in creating an enabling environment where social innovations can more effectively integrate into health systems to maximize their impact on beneficiaries.

**Main text:**

The integration of social innovations into health systems is essential to ensure their sustainability and the wide dissemination of their impact. Effective partnerships, strong engagement with and endorsement by governments and communities, regulations, trust and sometimes willingness are key factors to enhance system integration, replication and dissemination of the models. Three examples of social innovations selected by the Social Innovation in Health Initiative illustrate the importance of engaging with governments and communities in order to link, integrate and synergize their efforts. Key challenges that they encountered, and lessons learnt are highlighted. Multilateral organizations and governments increasingly engage in promoting and supporting the development, testing and dissemination of social innovations to address the health care delivery gap. They play an important role in creating an enabling environment. This includes promoting the concept of social innovation in health care delivery, spreading social innovation approach and lessons learnt, fostering partnerships and leveraging resources, convening communities, health system actors and various stakeholders to work together across disciplines and sectors, and nurturing capacity in countries.

**Conclusions:**

Multilateral organizations and local and national governments have a critical role to play in creating an enabling environment where social innovations can flourish. In supporting and disseminating social innovation approach, multilateral organizations and governments have a great opportunity to accelerate Universal Health Coverage and the achievement of the Sustainable Development Goals.

**Electronic supplementary material:**

The online version of this article (10.1186/s40249-019-0592-y) contains supplementary material, which is available to authorized users.

## Multilingual abstracts

Please see Additional file 1 for translations of the abstract into the five official working languages of the United Nations.

## Background

Despite great medical advances and scientific progress over the past century, one billion people globally still lack access to basic health care services. Research and development efforts on developing novel technologies, medicines, vaccines and diagnostics have failed to reach many populations, in particular those marginalized and in greatest need. “Leaving no one behind” is at the core of the 2030 Agenda for Sustainable Development and of the Sustainable Development Goals. These commitments call for innovative approaches [1] to provide quality, accessible and affordable healthcare. Social innovation in health models aim to provide solutions that enhance approaches to implementing health care programmes to bridge the health care delivery gap, address equity and create social value. This commentary highlights the role of multilateral organizations and governments in creating an enabling environment where social innovations can more effectively integrate within health systems to maximize their impact for beneficiaries. It presents three examples of social innovations illustrating the importance of engaging with governments and communities to link, integrate and synergize their efforts, some of the challenges encountered and lessons learnt.

## Main text

### Innovative solutions to improve health systems

Social innovation, as defined by Phills and co-authors, is “the process of inventing, securing support for, and implementing novel solutions to social needs and problems” [2]. As applied to healthcare access and coverage, the aim is to ensure that health systems and services are available to all people, including the most vulnerable and hard-to-reach populations. The integration of innovations into health systems is essential to ensure the sustainability and wide-spread dissemination of their impact [2]. Health systems include communities and actors from the public, private and civil society sectors. As highlighted in the 2030 Agenda for Sustainable Development [1], there is a need to break down boundaries between the different sectors to facilitate new interactions, build partnerships among the various health system actors, and foster the strong engagement of different communities. Lack of partnerships, engagement, endorsement, regulations, trust and sometimes willingness may prevent innovations from being replicated and disseminated. Social innovations offer a model to address this silo approach and promote an inclusive process engaging communities [3] and various health actors to work together to identify problems and develop and implement innovative solutions. They focus on the people they serve rather than on diseases, and promote multidisciplinary approaches and collaborations between various sectors.

### Learning from social innovations in low- and middle-income countries

Examples of social innovations in health exist in many parts of the world, even if these are not always identified as such. However, evidence of what works, what does not work, and lessons learnt, in particular in low- and middle-income countries, is not systematically collected, shared and discussed. Research is needed to better understand the factors involved in the feasibility, effectiveness and sustainability of innovations. Research is also needed for these innovations to achieve a wider impact, through better comprehending and describing the mechanisms for replication, potential scalability and dissemination [4].

In 2014 the Social Innovation in Health Initiative (SIHI) was established as a collaboration between TDR (the Special Programme for Research and Training in Tropical Diseases), the Bertha Centre for Social Innovation and Entrepreneurship at the University of Cape Town Graduate School of Business, the Skoll Centre for Social Entrepreneurship at Oxford University, and the London School of Hygiene and Tropical Medicine. The initiative aims to advance community-engaged social innovation in health within low- and middle-income countries, through fostering research, building capacity, and undertaking advocacy towards greater health equity through system integration. In 2016, the network expanded to engage low- and middle-income countries to establish SIHI country hubs through collaboration with Makerere University in Uganda, the University of Malawi, the University of the Philippines, and later, the Centro Internacional de Entrenamiento e Investigaciones Médicas in Colombia and the Social Entrepreneurship to Spur Health in China. These hubs promote the concept of social innovation and provide a platform to convene the various health system actors to identify problems and develop solutions. They foster research on social innovation and help to strengthen research capacity. The initiative also supports social innovations by strengthening the capacity of these organizations to sustain and replicate themselves, enhance cross-sectoral partnerships, and conduct research on how best to engage with governments. In addition, SIHI collaborates with various organizations advancing innovation in health and contributing to SIHI’s mission [5].

A first step for the Social Innovation in Health Initiative in 2015 was to identify and study selected cases of social innovations which were addressing health care delivery and had been operational for at least a year in a low- or middle-income country. Following an open call, 179 nominations were received and 23 cases across 15 countries were selected by an expert review panel. Selection criteria included appropriateness of the solution, degree of innovativeness, inclusiveness, affordability, effectiveness, scalability and sustainability. Research on these innovations was conducted to better understand how they had been developed and implemented, the opportunities and challenges they faced in delivering their services and in making and measuring their impact, the factors involved in their sustainability and potential scalability, and the main lessons learnt [6, 7].

The innovations studied used various types of business models to run their respective operations. The three examples described below highlight the importance of strong engagement with governments and health systems actors to sustain and replicate the impact of these organizations.

Sproxil, Inc. is a for-profit company established in 2009 and initially operating in Nigeria to build trust across supply chains of pharmaceutical products. The company provides a unique labelling system, combined with technology to identify counterfeit medicine at the point of purchase. The technology also enables data collection on counterfeit products, providing valuable insight for regulatory authorities and manufacturers. Sproxil, Inc. only charges manufacturers, who pay into the system for their products to be protected. However, it also receives donor grants to enable expansion in new countries. Within the first six years, Sproxil, Inc. had expanded from Nigeria to Ghana, India, Kenya, Pakistan and the United Republic of Tanzania, and registered more than 20 million authentications, with 12 million unique users of pharmaceutical products. The company turned profitable in 2014. One key element to sustain and replicate this model is endorsement from regulatory authorities. This enhances the confidence of both manufacturers and consumers and provides countries with critical data to protect against counterfeit products and to strengthen the supply chain. Challenges to expand include: (i) lack of mobile phone penetration and a reliable telecommunication network; (ii) level of engagement with government and regulatory authorities; and (iii) poor awareness of consumers of counterfeit products and their danger [6, 7].

A second example is One Family Health, a public-private community partnership in Rwanda. A foundation was established in 2012 in the United Kingdom of Great Britain and Northern Ireland in order to enhance access to essential primary care services for people living in mountainous rural areas of Rwanda. In partnership with the Rwandan Ministry of Health and an operating agreement with district level health departments, One Family Health established a limited liability low profit company to run franchised nurse-owned health posts. These private providers are integrated in the health system and leverage the national health insurance scheme to deliver sustainable care. As partners, the Ministry of Health and, when required, the community provide the land and infrastructure for health posts to be established. The health posts are financed by fees for service, while the One Family Health Foundation is financed through franchise royalty fees, marketing and pharmaceutical product sales. Within the first three years, One Family Health had opened more than 90 health posts which served more than 500 000 patients across 11 districts in Rwanda. The partnerships with government, community and other health system actors are key elements in the significant and sustainable impact of this approach and in the possibility of replicating the model in other African countries. Challenges that limit the speed of expansion include: (i) the need to ensure that health system actors trust and support the model; (ii) the need to invest in developing strong payment systems and operating procedures; and (iii) availability of government infrastructure in villages to establish health posts. Political and economic stability in the country, implementation of universal health coverage, and willingness of the government to engage in a public-private partnership are crucial elements for replication of this model in other countries [6, 7].

The third example is a nongovernmental organization, using a revenue generating business model to empower community members to become social entrepreneurs. Living Goods Uganda was established in 2007 and adopted good practice in entrepreneurship and performance management to deliver a community health worker programme which has been effective in reducing under five child mortality by 27% and neonatal mortality by 33% after three years of intervention [8]. Living Goods provides ongoing training, access to quality medicines and products, and performance incentives to village-based health entrepreneurs. Through revenue generation from product sales and franchised royalty fees, Living Goods is able to sustain 60% of its operations. The organization also receives unrestricted donor grants, which are used for expanding the model through public sector partnerships in other regions in Uganda and in other countries. In 2013 Livings Goods considered relying solely on their revenue. Yet becoming financially self-sustaining would have limited its scaling capacity and the number of lives saved. The organization opted to continue receiving donor grants to support its scale up. Critical elements of Living Goods’ scaling strategy are: (i) integration with the government; (ii) replication through partnerships with other actors; and (iii) leverage of large scale funding [6, 7].

These examples illustrate the potential value of developing not only strategies for organizational scaling-up, but also strategies to link and integrate the innovation into the health system to enhance sustainability and to create a wider impact reaching more beneficiaries. Developing public-private partnerships requires effort and time investment that might slow down the expansion of social innovations, yet this is critical to success and sustainability. These lessons confirm the findings from case studies in the report *Beyond Organizational Scale: How Social Entrepreneurs Create Systems Change* [9]. The report highlights how changing systems rules can require neutral organizations or institutions as “honest brokers.” When looking at social impact, the choice of the business model should be based on the dynamics of the market and context in which the organization operates.

Partnerships between public, private and philanthropic sectors emerged as an important factor for systemic change and sustainable impact. Too often engagement with governments seems to be a challenge. In the above case studies and in most of the other case studies documented, replicability and scalability of the social innovation approach in new countries strongly depend on the government’s willingness to engage in multisectoral partnerships. To support and enhance a systems approach, a change of mindset in approaching health care delivery is needed at various levels of the system. The value of engaging communities and working with experts from various disciplines and diverse sectors needs to be promoted at the global health level in order to create awareness and influence practices at national and local levels.

### Creating an enabling environment

Building upon the lessons learnt, in 2015 the World Health Organization made a call for global action to embrace and adopt social innovation [10]. The call invited governments, multilateral organizations and other health system actors to introduce innovative and effective approaches to enhance health care delivery and reach vulnerable populations. It promoted the value of social innovations in health care delivery with the aim to spread the approach in low- and middle-income countries.

Multilateral organizations and governments have a key role to play in fostering such change and in creating an enabling environment. They are increasingly engaged in promoting and supporting the development, testing and dissemination of social innovations and of their approach to address the health care delivery gap. Special initiatives and programmes include the Social Innovation in Health Initiative mentioned above [11]; The Innovation for Uptake, Scale and Equity in Immunisation programme launched by the Global Alliance for Vaccines and Immunisation, the Vaccine Alliance [12]; the *Innovation Facility* established at United Nations Development Programme (UNDP) [13]; and the UNICEF *Innovation Centre* [14]. The *WHO framework for people-centred integrated health services* provides a practical guide for social innovations. It calls for steps to unlock the capacity of people and communities to take an active role in their own health and the health system, to reorient health services to ensure that care is provided in the most appropriate settings, and to coordinate care across providers, organizations, care settings and beyond the health sector to include social and other relevant services [15].

Creating an enabling environment also involves generating and fostering new knowledge to better understand what works and what does not work, and how to sustain their efforts and their impact. There is a need to build capacity to integrate research in the processes of social innovation. Social innovators, community members and other health system actors need to engage in research to better understand how to improve performance, how to best engage with governments, and which factors are involved in moving towards the sustainability, replicability and scalability of their innovations as relevant.

Multilateral organizations can facilitate linkages to governments and can catalyse, through their normative role, the development of guidelines and standards to support health actors to advance social innovations and integrate research in their process. Multilateral organizations and local and national governments also have an important role to play to help mobilize resources for innovations through, for example, the creation of innovation funds (e.g. UNICEF Innovation Fund, UNDP Innovation Facility). They draw on and provide unlimited networks of experts at the global level and at the regional and local country office level. These foster an environment in which social innovations can thrive and flourish.

## Conclusions

Multilateral organizations and governments have a critical role to play in promoting the concept of social innovation in health care delivery, in spreading its approach, in nurturing social innovation organizations and in creating an enabling environment. They can advocate and promote the value of social innovations at local, national and global levels. They can actively support platforms to engage communities and bring all actors to work together. They can promote and support the integration of research in social innovations to better understand what works and what does not work. They can foster and build partnerships and leverage financial or in-kind resources and support capacity strengthening in countries. In supporting and disseminating social innovation approach in countries, multilateral organizations and governments have a great opportunity to learn and become more agile and responsive to accelerate Universal Health Coverage and the achievement of the Sustainable Development Goals.

## Additional file

Additional file 1: Multilingual abstracts in the five official working languages of the United Nations. (PDF 494 kb)

## Data Availability

Not applicable.

## Abbreviations

HIV: Human immunodeficiency virus;
SIHI: Social Innovation in Health Initiative
TDR: Special Programme for Research and Training in Tropical Diseases, cosponsored by the United Nations Children’s Fund, the United Nations Development Programme, the World Bank and the World Health Organization
UNDP: United Nations Development Programme
UNICEF: United Nations Children’s Fund
WHO: World Health Organization
