# Supplementary material for: The role of multilateral organizations and governments in advancing social innovation in health care delivery
Source: Infect Dis Poverty. 2019 Sep 13;8:81. doi: 10.1186/s40249-019-0592-y (PMC6743093; doi:10.1186/s40249-019-0592-y)
Supplement: Supplementary file 1 — Multilingual abstracts in the five official working languages of the United Nations. (PDF 494 kb) [file 40249_2019_592_MOESM1_ESM.pdf]

Translation of the abstract into the five official working languages of the United Nations

دور المنظَمات متعدُدة الأطراف والحكومات في النَهوض بالإبتكار الإجتماعي في تقديم الرُعاية الصحية

بياتريس هالباب، روزانا و. بيلينغ، فرانسوا بونيتشي

موجز

الخلفية: بالرَغم من التقدَم الطبي والتطوَر العلمي الكبير خلال القرن الماضي، لا يزال هناك مليار شخص في العالم يفتقرون إلى خدمات الرعاية الصحية الأساسية. في سياق خطة التَنمية المستدامة لعام 2030 ، تهدف نماذج الإبتكار الإجتماعي إلى توفير حلول فعَالة لسد فجوة قائمة في تقديم الرُعاية الصُحية، ومعالجة قضايا المساواة وخلق قيمة اجتماعية. تسلُط هذه الدُراسة الضَوء على دور المنظَمات متعدُدة الأطراف والحكومات في خلق بيئة مؤاتية حيث يمكن إدخال الإبتكارات الإجتماعية في الأنظمة الصحية بطريقة أكثر فعَالية من أجل مضاعفة تأثيرها إلى أقصى حد على المستفيدين.

النص الرئيسي: يعتبر دمج الإبتكارات الإجتماعية في الأنظمة الصحية أمرا " ضروريَا" لضمان استدامتها ونشر تأثيرها على نطاق واسع. إنَ كلَ من الشَراكات الفعَالة، والمشاركة القويَة مع الحكومات والمجتمعات وبتأييد منها، والأنظمة، والثقة وفي بعض الأحيان الإستعداد تعد جميعها عوامل أساسيَة لتعزيز تكامل النُظام، وتكرار النَماذج ونشرها. اختار الإبتكار الإجتماعي ضمن مبادرة الصحة هيلث إينيشياتيف ثلاثة أمثلة عن الإبتكارات الإجتماعية تبرز أهمُية التواصل مع الحكومات والمجتمعات من أجل ربط جهودها ودمجها وتضافرها فيما بينها. يتم تسليط الضَوء على التحدٌيات الرئيسيَة التي قاموا بمواجهتها، والدروس المستقاة منها. تنخرط المنظَمات متعدٌدة الأطراف والحكومات بشكل متزايد في ترويج ودعم تطوير، واختبار ونشر الإبتكارات الإجتماعية لمعالجة الفجوة القائمة في تقديم الرُعاية الصحية. هي تلعب دورا" هامَا"في خلق بيئة مؤاتية. ويشمل ذلك ترويج مفهوم الإبتكار الإجتماعي في تقديم الرُعاية الصحية، ونشر نهج الإبتكار الإجتماعي والدُروس المستقاة ، وتعزيز الشَراكات وتعبئة الموارد ، واجتماع المجتمعات، والجهات الفاعلة في النُظام الصحي ومختلف الأطراف المعنيَة للعمل معاً عبر التخصُصات والقطاعات ، وتنمية القدرات في البلدان.

النتائج: تلعب المنظَمات متعدٌدة الأطراف والحكومات المحلٌية والوطنيَة دورا" هاما"في خلق بيئة مواتية يمكن أن تزدهر فيها الإبتكارات الإجتماعية. تمتلك المنظَمات متعدٌدة الأطراف والحكومات في حملتها لدعم ونشر نهج الإبتكار الإجتماعي فرصة كيرى لتسريع التَغطية الصحٌية الشَاملة وتحقيق أهداف التَنمية المستدامة.

Translated from English version into Arabic by Aghilas Mihoub, revised by Reem Zakkar, through

TRANSLATORS
WITHOUT BORDERS

多边组织和政府在推动卫生服务供给侧社会创新的作用

Beatrice Halpaap, Rosanna W. Peeling, Fran çois Bonnici

摘要

引言：尽管一个世纪以来我们在医学创新和科技方面取得了伟大的成就，但全球仍有 10 亿人无法获得基本的卫生服务保障。在 2030 年可持续发展议程框架下，社会创新模式为此提供了一个有效的解决方案，有助于弥补卫生服务供给侧差距、促进社会公平并创造社会价值。本述评突出了多边组织和各国政府在营造社会创新更有效地融入卫生体系的有利环境方面所发挥的作用，从而最大限度地使其发挥对受益者的影响。

正文：将社会创新融入卫生体系对确保其可持续性和扩大影响至关重要。有效的伙伴关系、政府和社区的大力参与和支持、规章制度、互相信任以及个人意愿是促进系统集成、可复制和模型推广的关键因素。社会健康倡议的社会创新组织（SIHI）选出的 3 个社会创新实例说明了政府和社区参与在加强联系、融合和协同方面的重要性，并重点介绍了面临的挑战和取得的经验教训。多边组织和各国政府越来越多地参与促进和支持社会创新的发展、试验和推广，以解决卫生服务供给侧的差距。它们在创造多种有利环境方面发挥重要作用，包括，在

卫生服务提供中推广的社会创新理念、传播社会创新方法和经验、促进伙伴关系和资源利用、社区参与、卫生体系执行者和各利益相关方进行跨学科和部门合作以及培养国际合作能力。
结论：多边组织与地区及国家政府在营造一个促进社会创新蓬勃发展的环境方面发挥着关键作用。在支持和推广社会创新过程中，多边组织和各国政府在加快全民健康覆盖和实现可持续发展目标方面面临重大机遇。

Translated from English version into Chinese by Peng Song, edited by Pin Yang

Le rôle des organisations multilatérales et des gouvernements dans la promotion de l’innovation sociale dans la prestation des soins de santé

Beatrice Halpaap, Rosanna W. Peeling, François Bonnici

Résumé
Contexte: malgré les grands progrès de la médecine et de la science au cours du siècle dernier, un milliard de personnes dans le monde n’ont toujours pas accès aux services de santé de base. Dans le cadre de l’agenda 2030 du Programme de développement durable, les modèles d’innovation sociale visent à fournir des solutions efficaces qui comblent l’écart dans la prestation des soins de santé, remédient à l’iniquité et créent une valeur sociale. Le présent commentaire souligne le rôle des organisations multilatérales et des gouvernements dans la création d’un environnement favorable où les innovations sociales peuvent s’intégrer plus efficacement dans les systèmes de santé afin d'optimiser leur impact sur les bénéficiaires.
Texte principal: l’intégration des innovations sociales dans les systèmes de santé est essentielle pour assurer leur pérennité et la large diffusion de leur impact. Des partenariats efficaces, un engagement ferme et le soutien des gouvernements et des communautés, des règlements, de la confiance et parfois la volonté de les appuyer sont des facteurs clés pour améliorer l’intégration des systèmes, la reproduction et la diffusion des modèles. Trois exemples d'innovations sociales choisies par l’Initiative d'innovation sociale dans le domaine de la santé illustrent l’importance de collaborer avec les gouvernements et les communautés afin de lier, d’intégrer et de synergiser leurs efforts. Les principaux défis qu’ils ont rencontrés et les enseignements tirés sont mis en évidence. Les organisations multilatérales et les gouvernements s'engagent de plus en plus à promouvoir et à appuyer l'élaboration, la mise à l'essai et la diffusion d'innovations sociales afin de combler l'écart dans les prestations de soins de santé. Ils jouent un rôle important dans la création d’un environnement favorable. Il s’agit notamment de promouvoir le concept d’innovation sociale dans la prestation des soins de santé; de diffuser l’approche d'innovation sociale et les enseignements tirés; de favoriser les partenariats et de mobiliser des ressources; de réunir les communautés, les acteurs du système de santé et les diverses parties prenantes pour qu’ils collaborent dans les disciplines et les secteurs; et de renforcer les capacités dans les pays.
Conclusions: les organisations multilatérales et les gouvernements locaux et nationaux ont un rôle essentiel à jouer dans la création d'un environnement favorable où les innovations sociales peuvent prospérer. En soutenant et en diffusant l’approche de l’innovation sociale, les organisations multilatérales et les gouvernements ont une excellente occasion d’accélérer la couverture (sanitaire) universelle et la réalisation des objectifs de développement durable.

Translated from English version into French by Bettina Stefan, revised by Johanne Arbour, through

Роль многосторонних организаций и государственных органов в продвижении социальных инноваций в области оказания медицинской помощи

Беатрис Галпаап, Розанна Пилинг, Франсуа Бонни

Аннотация

Справка: Несмотря на достижения медицины и научный прогрес за прошедшие 100 лет, один миллиард людей все еще остаются без доступа к основным медицинским услугам. В рамках "Повестка дня в области устойчивого развития на период до 2030 года", модели социальной инновации нацелены на получение эффективных решений, которые устранят пробел в области оказания медицинской помощи, установят равенство и создадут социальную значимость. Данная работа подчеркивает роль многосторонних организаций и государственных органов в создании благоприятной среды, в которой социальные инновации смогут эффективнее интегрировать в системы здравоохранения, чтобы принести максимальную пользу нуждающимся.

Основной текст: Интеграция социальных инноваций в системы здравоохранения необходимо для гаранитии их устойчивости и широкого распространения их влияния. Эффективное сотрудничество, сильная поддержка со стороны государства и общества, регулирующие правила, доверие, порой и желание, являются ключевыми факторами в расширении системы интеграции и воспроизведении моделей. Три примера социальных инноваций, выбранных "Социальными инновациями в области здравоохранения", свидетельствуют о необходимости сотрудничества как с государственными органами, так и с сообществами для интеграции и проталкивания собственных действий. Здесь изложены ключевые проблемы, с которыми пришлось столкнуться, и извлеченные из них уроки Многосторонние организации и государственные органы все чаще поддерживают развитие, испытание и применение социальных инноваций с целью устранения недостатка в области здравоохранения. Они играют важную роль в создании благоприятной среды. Сюда входят: продвижение концепции социальных инноваций в области оказания медицинской помощи, распространение социально-инновационных подходов и полученных знаний, поощрение сотрудничества и использование ресурсов, привлечение местного населения, структур системы здравоохранения и различных акционеров к совместной работе над рядом правил и секторами, а также содействие развитию стран.

Выводы: Многосторонние организации, местные и международные государственные органы играют ключевую роль в создании благоприятной среды для содействия процветанию социальных инноваций. Содействуя и расширяя социально-инновационный подход, многосторонние организации и государственные органы получают большую возможность для ускорения"Глобального распространения услуг здравоохранения" и достижения "Целей устойчивого развития".

Translated from English version into Russian by Gunel Huseynbayova, revised by Kenul Aliyeva-Aqarzayeva, through

TRANSLATORS WITHOUT BORDERS

El papel de los gobiernos y los organismos multilaterales en el progreso de la innovación social en la prestación de atención sanitaria

Beatrice Halpaap, Rosanna W. Peeling, François Bonnici

Resumen

Contexto: A pesar de los grandes avances médicos y el progreso científico que ha tenido lugar a lo largo del pasado siglo, mil millones de personas en todo el mundo aún no tienen acceso a servicios básicos de atención sanitaria. En el contexto de la Agenda 2030 para el Desarrollo Sostenible, los modelos de innovación social tienen como objetivo proporcionar soluciones efectivas que cubran la brecha que existe en la prestación de atención sanitaria, aborden la equidad y creen valor social. Este comentario destaca el papel que juegan los gobiernos y los organismos multilaterales en la creación de un entorno favorable donde las innovaciones sociales puedan integrarse de manera más efectiva en los sistemas sanitarios para aprovechar al máximo su impacto en los beneficiarios.

Texto principal: La integración de innovaciones sociales en los sistemas sanitarios resulta esencial para garantizar su sostenibilidad, así como una amplia difusión de su impacto. Las alianzas efectivas, un fuerte compromiso y respaldo por parte de los gobiernos y las comunidades, regulaciones, confianza y, en ocasiones, la voluntad son factores clave para mejorar la integración del sistema, la réplica y la difusión de los modelos. Tres ejemplos de innovaciones sociales seleccionadas por la Iniciativa de Innovación Social en Salud ilustran la importancia de involucrar a los gobiernos y las comunidades para vincular, integrar y sinergizar sus esfuerzos. Se destacan los desafíos clave que encontraron y las lecciones que aprendieron. Los organismos multilaterales y los gobiernos se dedican cada vez más a fomentar y apoyar el desarrollo, las pruebas y la difusión de innovaciones sociales para abordar la brecha que existe en cuanto a la prestación de servicios sanitarios. Desempeñan un papel clave en la creación de un entorno favorable. Esto incluye fomentar el concepto de innovación social en la prestación de servicios sanitarios, difundir el enfoque de innovación social y las lecciones que han aprendido, fomentar las alianzas y aprovechar los recursos, involucrar a las comunidades, al sistema sanitario y a las diversas partes interesadas para así trabajar juntos en todas las disciplinas y sectores, y fomentar desarrollo en los países.

Conclusiones: Los organismos multilaterales y los gobiernos tanto locales como nacionales desempeñan un papel fundamental en la creación de un entorno propicio donde las innovaciones sociales puedan florecer. Al apoyar y difundir el enfoque de innovación social, los organismos multilaterales y los gobiernos se encuentran con una gran oportunidad para acelerar el proceso de conseguir un seguro sanitario universal y lograr los Objetivos de Desarrollo Sostenible.

Translated from English version into Spanish by Noemí Jiménez, revised by Laura García Sánchez, through

TRANSLATORS
WITHOUT BORDERS
